# Serum Resistin Level and Progression of Atherosclerosis during Glucocorticoid Therapy for Systemic Autoimmune Diseases

**DOI:** 10.3390/metabo6030028

**Published:** 2016-09-16

**Authors:** Nahoko Tanaka, Shotaro Masuoka, Natsuko Kusunoki, Toshihiro Nanki, Shinichi Kawai

**Affiliations:** Division of Rheumatology, Department of Internal Medicine, School of Medicine, Faculty of Medicine, Toho University, Tokyo 143-8541, Japan; nahoko@med.toho-u.ac.jp (N.T.); shoutarou.masuoka@med.toho-u.ac.jp (S.M.); kusunoki@med.toho-u.ac.jp (N.K.); toshihiro.nanki@med.toho-u.ac.jp (T.N.)

**Keywords:** adipokines, resistin, atherosclerosis, glucocorticoid, systemic autoimmune diseases

## Abstract

Adipokines are important regulators of several processes, including inflammation and atherosclerosis. In patients with systemic autoimmune diseases, atherosclerosis is accelerated with higher cardiovascular morbidity and mortality. We prospectively investigated the association of adipokines and glucocorticoid therapy with progression of premature atherosclerosis in 38 patients starting glucocorticoid therapy for systemic autoimmune diseases. To detect premature atherosclerosis, carotid ultrasonography was performed at initiation of glucocorticoid therapy and after a mean three-year follow-up period. The ankle-brachial pressure index and cardio-ankle vascular index (CAVI) were measured. Serum adipokine levels were determined with enzyme-linked immunosorbent assay kits. Twenty-three patients (60.5%) had carotid artery plaque at baseline. The carotid artery intima-media thickness (IMT) increased significantly during follow-up. Glucocorticoids reduced the serum resistin level, while increasing serum leptin and high molecular weight-adiponectin. There was slower progression of atherosclerosis (carotid IMT and CAVI) at follow-up in patients with greater reduction of serum resistin and with higher cumulative prednisolone dose. In conclusion, progression of premature atherosclerosis occurred at an early stage of systemic autoimmune diseases before initiation of glucocorticoid therapy. Since resistin, an inflammation and atherosclerosis related adipokine, is reduced by glucocorticoids, glucocortidoid therapy may not accelerate atherosclerosis in patients with systemic autoimmune diseases.

## 1. Introduction

Adipose tissue synthesizes and releases various physiologically active molecules that are known as adipokines or adipocytokines, including resistin, leptin, and adiponectin, as well as interleukins (IL-1, IL-1 receptor antagonist, IL-6, and IL-10) and tumor necrosis factor (TNF)-α [1]. Adipocytes have an established important role in regulating the systemic energy balance and glucose homeostasis [2]. More recently, it has been suggested that they adipokines are important regulators of several processes including immunity and inflammation, and may even play a role in atherosclerosis [1,3].

Patients with systemic autoimmune diseases show accelerated development of atherosclerosis with an increased risk of cardiovascular morbidity and mortality compared to the general population [4,5,6,7,8,9,10,11]. Traditional cardiovascular risk factors, such as hyperlipidemia, hypertension, Ődiabetes mellitus, smoking and aging do not always explain this increased risk of cardiovascular disease (CVD) associated with systemic autoimmune diseases [12,13,14]. Therefore, it has been suggested that autoimmunity/inflammation might contribute to accelerated atherosclerosis in these patients. However, the underlying mechanisms of accelerated atherosclerosis remain unclear, and the impact of medications such as glucocorticoids on atherosclerosis is still controversial.

We have previously reported that adipokines, especially resistin, may be associated with inflammatory processes in rheumatoid arthritis (RA) [15], Kawasaki disease [16], and other systemic autoimmune diseases [17]. Glucocorticoid therapy rapidly reduces elevated resistin levels to the normal range over four weeks in patients with active systemic autoimmune diseases [17]. In addition, several studies have suggested that resistin may play a role in atherosclerosis [1,3]. We hypothesized that glucocorticoids could have an antiatherosclerotic effect in patients with systemic autoimmune diseases by improving hyper-resistinemia. In the present study, we performed a longitudinal investigation into the association between changes of adipokine levels and progression of premature atherosclerosis over a three-year follow-up period in patients newly starting glucocorticoid therapy for systemic autoimmune diseases. The influence of glucocorticoid therapy on premature atherosclerosis was also investigated.

## 2. Results

### 2.1. Patient Profile

The baseline characteristics of the subjects are shown in Table 1. There were 38 patients with recent-onset systemic autoimmune diseases (11 men and 27 women) and the median disease duration was 4.4 weeks. These 38 patients underwent carotid ultrasonography at baseline and at follow-up after receiving glucocorticoid therapy for an average of 3.3 ± 0.9 years (mean ± standard deviation (SD)).

Clinical and laboratory data of the patients obtained at baseline and at follow-up are shown in Table 2. All patients had recent-onset active disease with no prior immunosuppressive therapy including glucocorticoids at baseline. The mean initial daily dose of prednisolone was 48.2 ± 9.0 mg. At follow-up, the mean daily dose of prednisolone was 7.9 ± 9.9 mg and the mean cumulative dose of prednisolone was 18,651 ± 8734 mg. The diastolic blood pressure, serum levels of total cholesterol (T-chol) and high-density lipoprotein cholesterol (HDL-chol), prevalence of diabetes mellitus, and use of antihypertensive agents, antidiabetic agents and statins were all significantly increased at follow-up, while the prevalence of smoking and the serum level of C-reactive protein (CRP) were decreased. No new CVD events occurred during the follow-up period.

### 2.2. Serum Adipokines

**Figure 1:**
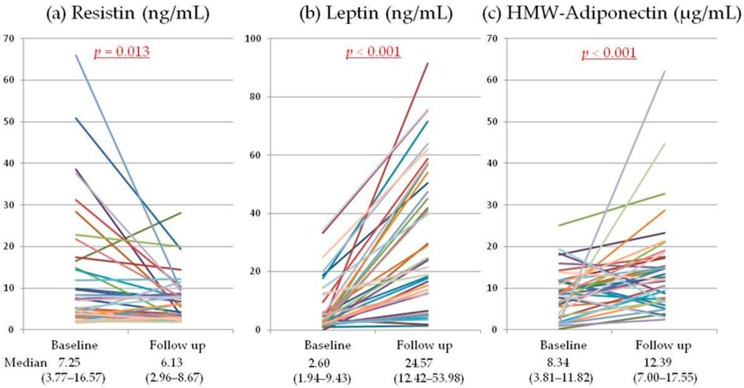
Changes of serum adipokine levels: (**a**) The median serum resistin level decreased with glucocorticoid therapy (from 7.3 to 6.1). (**b**) In contrast, the serum leptin level (from 2.6 to 24.6) increased; as did (**c**) the serum HMW-adiponectin level (from 8.3 to 12.4). Median values (interquartile range) are shown under the graphs. Significant differences compared with baseline (underlined) were determined by the Wilcoxon singed-rank sum test. HMW, High Molecular Weight.

We examined whether glucocorticoid therapy affected serum adipokine levels in our patients with systemic autoimmune diseases. Compared with baseline, the serum level of resistin showed a significant decrease after three years of glucocorticoid therapy, while the serum levels of leptin and high molecular weight (HMW)-adiponectin both increased significantly (Figure 1).

### 2.3. Premature Atherosclerosis

Twenty-three of the 38 patients (60.5%) had carotid artery plaque at baseline. Among the 15 patients without plaque, two patients (5.3%) developed new plaque during the follow-up period. The median value of the maximum carotid artery intima-media thickness (IMT) increased significantly from 0.675 (IQR: 0.500–0.813) mm at baseline to 0.725 (0.588–0.725) mm at follow-up (*p* = 0.04 vs. the null hypothesis of 0.000 mm annual change). Among the 38 patients, the median IMT showed an annual increase of 0.016 (0.026–0.067) mm. An increase of IMT was observed in 21 patients (55.3%), while IMT was unchanged or decreased in 17 patients (44.7%). Baseline clinical and laboratory data were similar between patients with and without progression of IMT.

The mean cardio-ankle vascular index (CAVI) was 7.8 ± 1.5 (SD) and the mean ankle brachial index (ABI) was 1.2 ± 0.1 at follow-up. None of the patients had symptoms or signs of peripheral arterial obstruction, except for one patient aged 75 with polymyositis and antiphospholipid syndrome (APS) who had intermittent claudication. However, five of the 38 patients (13.2%) had an abnormal ABI (ABI < 1.0) according to the consensus statement [18]. The mean cumulative dose of prednisolone was significantly higher in the patients with a normal ABI than in those with an abnormal ABI (*p =* 0.038). There were no significant differences between the normal and abnormal ABI groups with regard to the serum concentrations of adipokines at baseline and follow-up or the annual changes of any adipokine during the follow-up period.

### 2.4. Multivariate Analysis of Factors Associated with Progression of Premature Atherosclerosis

We next examined the independent influence of prednisolone or serum adipokines (resistin, leptin, and HMW-adiponectin) on the progression of premature atherosclerosis in our patients with systemic autoimmune diseases by multiple regression analyses adjusted for patient characteristics (gender, age, and BMI), traditional risk factors (hypertension, diabetes mellitus, smoking status, and serum levels of HDL-chol, triglycerides (TG) and CRP), and a history of CVD.

Multivariate analysis showed that the median annual change of the maximum carotid artery IMT over the follow-up period was positively associated with the annual change of the serum resistin level. In contrast, the median annual change of the maximum carotid artery IMT was negatively associated with cumulative prednisolone exposure. None of traditional risk factors was independently associated with the increase of IMT (Table 3, multivariate model).

According to multiple regression analysis, the CAVI at follow-up was independently associated with the annual change of the serum resistin level, in addition to age and diabetes mellitus (Table 4, multivariate model). In contrast, multiple regression analysis showed that the ABI at follow-up was only associated with a history of CVD. There was no association between the ABI and the serum levels of any of the adipokines or the cumulative prednisolone dose.

## 3. Discussion

In this study, we demonstrated that premature atherosclerosis was already progressing in patients with systemic autoimmune diseases at an early stage before initiation of glucocorticoid therapy. We also found slower progression of premature atherosclerosis (evaluated from the carotid IMT and CAVI) in patients with greater reduction of the serum resistin level at follow-up and patients receiving higher cumulative dose of prednisolone. The change of the serum resistin level was positively associated with the increase of IMT and CAVI, suggesting that resistin might play a role in the progression of atherosclerosis associated with systemic autoimmune diseases. Since resistin, an inflammation and atherosclerosis related adipokine, is reduced by glucocorticoids, glucocortidoid therapy may not accelerate atherosclerosis in patients with systemic autoimmune diseases.

Assessment of carotid artery plaque and the IMT by ultrasonography is a noninvasive and reliable method for assessing the systemic burden of atherosclerosis [19]. Carotid atherosclerosis can be used as a surrogate marker for coronary atherosclerosis and is a good predictor of future cardiovascular events in the general population. Investigation of carotid artery plaque and the IMT has also been suggested to be useful for predicting cardiovascular events in patients with RA [20] and patients with systemic lupus erythematosus (SLE) [21].

The prevalence of carotid artery plaque was 19.3% in a large sample of relatively elderly volunteers (mean age: 59–70 years) [22]. Our 38 patients (mean age: 49.3 years) showed a higher prevalence of carotid artery plaque before initiation glucocorticoid therapy (60.5%) compared with that in the older general population. These findings seem to be compatible with a previous report about a similar prevalence of carotid artery plaque in patients with SLE (51.9%; mean age: 52 years) [23]. The median IMT of our patients (0.68 mm; mean age: 49.3 years) was also consistent with that reported for SLE patients (0.62 mm; mean age: 52 years) [23]. Moreover, a significant increase of the carotid IMT in rheumatic diseases (including RA, SLE, and systemic sclerosis) has been reported compared with healthy control subjects [24]. Most of the subjects enrolled in previous cross-sectional studies were outpatients with long disease duration who were already on various therapies, so the impact of specific medications such as glucocorticoids on atherosclerosis has not been clarified.

In this study, we identified the progression of premature atherosclerosis in patients with recent-onset systemic autoimmune diseases before initiation of glucocorticoid therapy. It had been reported that traditional risk factors (such as hyperlipidemia, hypertension, diabetes mellitus, smoking, and age) do not always explain the increased risk of cardiovascular disease associated with systemic autoimmune diseases [12,13,14]. Ross [25] described atherosclerosis as a chronic inflammatory disease affecting large and medium-sized elastic and muscular arteries, and stated that inflammation plays a role in all stages of atherosclerosis. Both inflammatory and immune processes are now recognized to play a role in accelerated atherosclerosis and the occurrence of cardiovascular events in patients with systemic autoimmune diseases [13,26]. The European League Against Rheumatism (EULAR) Task Force for cardiovascular risk management has recommended adequate control of disease activity to lower the cardiovascular risk in patients with RA and inflammatory arthritis [27].

As clinical manifestations of atherosclerosis, cardiovascular events are observed before or soon after the diagnosis of SLE and RA [28,29]. Subclinical autoimmune/inflammatory processes before the onset of overt disease may lead to the development of premature atherosclerosis [30], which was detected by carotid ultrasonography in our patients at diagnosis.

We found that 13.2% of our patients (mean age: 49.3 years) had an abnormal ABI (ABI < 1.0), although there were no symptoms or signs of peripheral arterial obstruction, except in one patient with polymyositis and APS. The ABI is a well-established and reproducible method with a high sensitivity and specificity for assessing the patency of lower limb arteries and detecting peripheral arterial disease [18]. It has been demonstrated to be a strong predictor of cardiovascular events in patients with peripheral arterial disease [31]. In addition, a low ABI is a marker of generalized atherosclerosis, and even asymptomatic reduction of the ABI (< 1.0) is inversely associated with an increased risk of atherosclerotic CVD in the general population [32,33]. In the middle-aged general population (mean age: 53–55 years), the prevalence of an ABI ≤ 0.9 is reported as under 4% [34], while the prevalence of an abnormal ABI increases rapidly with age in the elderly population [32,33]. Thus, the prevalence of an abnormal ABI was higher in our patients compared with the general population of a similar age.

CAVI is a recently developed index of arterial stiffness that is calculated from the heart-ankle pulse wave velocity (haPWV) adjusted for blood pressure by β, a stiffness parameter. Therefore, CAVI represents the stiffness of the arterial tree from of the origin the aorta to the ankle, and is not influenced by blood pressure changes during measurement [35]. In addition to being a reproducible marker of early atherosclerosis, CAVI was recently reported to be a predictor of future atherosclerotic cardiovascular events [36,37,38]. CAVI is also associated with coronary arteriosclerosis, and the cut-off point for the presence of coronary stenosis was reported to be 8.8 [39]. In this study, six of the 38 patients with systemic autoimmune diseases (15.7%) had a CAVI > 8.8. Moreover, the mean CAVI value in our patients (7.8; mean age: 49.3 years) was comparable to that in patients of a similar age with metabolic syndrome (7.9; mean age: 53.8 years), which is a well-established cardiovascular risk factor. It was also reported that patients with metabolic syndrome and higher CAVI values have a higher frequency of cardiovascular events and myocardial infarction [36].

Resistin was originally identified as a 12.5 kDa polypeptide expressed and secreted by white adipose tissue that was reported to induce insulin resistance in rodents [40]. In contrast to the findings in animal models, adipocytes express resistin at very low levels or not at all in humans, whereas there is high expression by peripheral blood mononuclear cells (PBMCs) (especially, monocytes and T lymphocytes), macrophages, neutrophils, and bone marrow cells involved in the inflammatory response [41,42,43,44]. Previous studies have demonstrated pro-inflammatory properties of resistin, which stimulated the synthesis and secretion of pro-inflammatory cytokines such as TNF-α and IL-6, and activated the transcription factor NF-κB in adipocytes and mononuclear cells. It was also reported that resistin is up-regulated by TNF-α, IL-1 β, IL-6, and lipopolysaccharide (LPS) [45,46].

Moreover, resistin is involved in the pathological processes leading to atherosclerosis and CVD, which are increasingly recognized as inflammatory conditions [1,3]. In vitro studies have indicated that resistin aggravates atherosclerosis by inducing vascular inflammation through stimulation of monocytes, endothelial cells, and vascular smooth muscle cells (VSMCs). In a clinical study, the plasma resistin level was associated with coronary atherosclerosis independently of metabolic syndrome and CRP [47]. Resistin has also been shown to predict the incidence of atherosclerotic CVD and CVD-related mortality in several general population studies [48,49,50].

We have previously reported that resistin may be associated with the inflammatory process in patients with systemic autoimmune diseases [17]. Serum resistin level was showed initially high in the patients with active systemic autoimmune diseases compared with the healthy controls, and it was decreased the normal range after glucocorticoid therapy [17]. In this study, multivariate analysis showed that the annual change of the serum resistin level was significantly associated with the annual change of the carotid IMT and CAVI, indicating that resistin might be associated with progression of premature atherosclerosis in systemic autoimmune diseases. Overall, our study suggested that resistin could represent a novel link between autoimmune-mediated inflammation and atherosclerosis in patients with systemic autoimmune diseases.

Chronic glucocorticoid therapy promotes certain cardiovascular risk factors, such as hypertension, insulin resistance, and hyperlipidemia [51], which could be indirectly linked to an increase of atherosclerotic cardiovascular events. In fact, glucocorticoid therapy has generally been reported to be associated with an increased risk of cardiovascular events in patients with RA, including myocardial infarction, stroke, and heart failure [52].

On the other hand, most studies of patients with SLE have shown that the daily glucocorticoid dose, cumulative dose, or administration period was not associated with the presence or progression of indicators of premature atherosclerosis, such as coronary artery calcification [53], carotid artery plaque, and the IMT [54,55,56]. Roman et al. [54] reported that patients with plaque were less likely to have received glucocorticoids than patients without plaque, and the average glucocorticoid dose was also lower in patients with plaque. Most of the recent cohort studies on SLE have shown that glucocorticoid therapy did not increase the risk of cardiovascular events [57,58,59,60,61], with one exception [62], although older cohort studies from 1990s found a relationship between glucocorticoid therapy and cardiovascular risk [9,63].

Anti-atherosclerotic effects of glucocorticoids have been demonstrated in animal models. Glucocorticoids prevent atherosclerosis in fat-fed rabbits, despite increasing hyperlipidemia [64,65]. The anti-atherosclerotic effect of glucocorticoids may depend on inhibition of inflammatory cell proliferation and foam cell formation [64,65], inhibition of intimal vascular smooth muscle cell (VSMC) proliferation [66], or reduced chemotactic attraction of circulating monocytes and macrophages into the sub-endothelial space [67].

Thus, glucocorticoids have a complex relationship with cardiovascular risk, since these drugs are effective for inhibiting inflammation and proliferation in atherosclerosis, but conversely can indirectly aggravate systemic cardiovascular risk factors. In addition, it is difficult to differentiate the influence of glucocorticoids from that of the underlying inflammatory condition, especially in patients undergoing treatment for autoimmune diseases. In large-scale general population studies, the mean annual progression of IMT was shown to be 0.001–0.010 mm [68,69]. In the present study, IMT increased by 0.016 mm/year during the follow-up period and progression of IMT was more rapid in our patients compared to the general population. However, it was negatively associated with cumulative exposure to prednisolone. The cumulative dose of prednisolone was significantly higher in the patients with a normal ABI than that in those with an abnormal ABI at follow-up. Regarding traditional cardiovascular risk factors, we found that the body mass index (BMI), diastolic blood pressure, prevalence of diabetes mellitus, and T-chol and HDL-chol levels were increased at follow-up after several years of glucocorticoid therapy for systemic autoimmune diseases. However, the changes of these risk factors were not significantly associated with progression of IMT. Therefore, it is suggested that aggressive glucocorticoid therapy might prevent acceleration of atherosclerosis due to inflammatory and immune-mediated processes in patients with active autoimmune diseases.

Furthermore, slower progression of premature atherosclerosis (evaluated by CAVI and carotid IMT) was found in patients with greater reduction of the serum resistin level after a mean follow-up period of three years. We previously reported that resistin expression was down-regulated by glucocorticoids both in vivo study in patients with active systemic autoimmune diseases and in vitro study using LPS-induced PBMCs [17]. The promoter region of the human resistin gene contains binding sites for proinflammatory transcription factors such as cRel (one of five NF-κB subunits) and AP-1, but has no glucocorticoid response element (GRE) [70]. Accordingly, glucocorticoid therapy might inhibit resistin expression by repressing activation of these transcription factors via the glucocorticoid receptor [71]. In contrast, Sasayama et al. [72] reported that glucocorticoids increased the resistin mRNA and protein expression of in patients without inflammatory conditions. Taken together with the suppression of hyper-resistinemia, related with inflammation and atherosclerosis, in active systemic autoimmune diseases by glucocorticoid therapy, it seems that glucocorticoids may not accelerate atherosclerosis.

## 4. Materials and Methods

### 4.1. Patients

This study was approved by the Ethics Committee of Toho University Medical Center Omori Hospital (approval number: 19-67, 24-77, and 24-96). The patients all gave written informed consent and were studied at Toho University Medical Center Omori Hospital.

This was a prospective observational study of 38 patients with systemic autoimmune diseases, including SLE (*n* = 16), vasculitis syndrome (*n* = 6), polymyositis/dermatomyositis (*n* = 14) and adult onset Still’s disease (*n* = 2). All subjects started glucocorticoid therapy (prednisolone at ≥ 30 mg daily) as inpatients of Toho University Omori Hospital. Patients were excluded if they had previously taken glucocorticoids or other immunosuppressive drugs, as were patients with large vessel vasculitis, such as Takayasu arteritis or giant cell arteritis, which often affect the carotid arteries [73].

### 4.2. Clinical and Laboratory Measurements

Clinical information and laboratory data were obtained from a structured interview, self-reported questionnaires, physical examination, and blood tests. BMI was calculated from the measured height and weight. Baseline blood pressure was determined as the average of two measurements. After an overnight fast, blood was collected in the morning to measure the baseline serum level of CRP by latex nephelometry (Eiken Chemical Co., Ltd., Tokyo, Japan), T-chol by the cholesterol dehydrogenase/ultraviolet method (Sysmex Corporation, Kobe, Japan), HDL-chol by the homogeneous method (Sekisui Medical Co., Ltd., Tokyo, Japan), and TG by an enzymatic assay (Sekisui Medical Co., Ltd., Tokyo, Japan). Low-density lipoprotein cholesterol (LDL-chol) was calculated by the formula of Friedewald et al. [74]. We assessed the smoking status, the presence or absence of hypertension (defined as a blood pressure ≥140/90 mmHg or use of antihypertensive medications), and the presence or absence of diabetes mellitus (defined according to Japan Diabetes Society criteria [75] or as the use of antidiabetic medications) as traditional risk factors for atherosclerosis.

### 4.3. Mesurement of Serum Adipokines

Fasting serum samples were collected before the start of glucocorticoid therapy (baseline) and were stored at −80 °C. Serum levels of adipokines were measured with enzyme-linked immunosorbent assay (ELISA) kits (resistin and leptin, B-Bridge International, Inc., Sunnyvale, CA, USA; HMW-adiponectin, Otsuka Pharmaceutical Co., Ltd., Tokyo, Japan).

### 4.4. Carotid Ultrasonography

To detect premature atherosclerosis, the carotid arteries were examined by ultrasonography according to the procedure described by Kumeda et al. [76] with some modifications, either before glucocorticoid therapy or within one month after starting therapy. Carotid ultrasonography was also conducted in all patients after a mean follow-up period of 3 years. In brief, the bilateral proximal common carotid artery, distal common carotid artery, carotid bulb, and internal carotid artery were examined with a Xario (SSA-660A) ultrasound diagnostic system (Toshiba Medical Systems Corporation, Ohtawara, Japan). Plaque was defined as a focal protrusion >1.1 mm thick in the walls of any of the above-mentioned arteries. The IMT was measured separately in the proximal and distal right and left common carotid arteries. Then the maximum IMT was obtained for each patient by averaging the maximum measurements for the right and left sides.

### 4.5. Measument of CAVI and ABI

At follow-up, CAVI and ABI were recorded using a VaseraVS-1000 vascular screening system (Fukuda Denshi, Tokyo, Japan) by the method of Shirai et al. [35]. The electrocardiogram, phonocardiograph, and pressures and waveforms of the brachial and ankle arteries were measured, and CAVI was automatically calculated by software in the apparatus. CAVI values were obtained by substituting the stiffness parameter β, recognized as a blood pressure-independent parameter of arterial stiffness, in the following equation for vascular elasticity and pulse wave velocity (PWV): β = 2ρ − 1/(Ps − Pd) × ln (Ps/Pd) − PWV^2^ (ρ is blood density, where Ps and Pd are SBP and DBP (in mmHg)). The right and left CAVI values were averaged for use in analysis. ABI was calculated as the highest ankle systolic pressure divided by the highest brachial systolic pressure on both sides, and the right and left measurements were averaged for use in subsequent analysis.

### 4.6. Statistical Analysis

Results are expressed as the mean ± standard deviation (SD) for normally distributed continuous variables or as the median with interquartile range (IQR) for continuous variables with a skewed distribution. Significant differences of background data between subgroups of patients were evaluated by Student’s *t*-test for normally distributed continuous variables and by the Mann–Whitney U test for continuous variables with a skewed distribution. Categorical variables were compared by the chi-square test or Fisher’s exact test. The significance of changes in serum adipokine levels, IMT, and other clinical data between baseline and follow-up was investigated by the paired-samples *t*-test or Wilcoxon signed ranks test for continuous variables, and by McNemar’s test for categorical variables. Simple linear regression was performed to assess correlations between IMT, CAVI, or ABI and patient characteristics. Stepwise forward multiple regression analysis was employed for multivariate analysis. Non-numerical variables were analyzed as categorical variables in the regression model. All statistical analyses were performed using StatFlex software (ver. 6; ARTEC Co., Ltd., Osaka, Japan). A two-sided probability of less than 0.05 was taken to indicate statistical significance.

## 5. Conclusions

The progression of premature atherosclerosis occurred at an early stage of systemic autoimmune diseases before initiation of glucocorticoid therapy. Since resistin, an inflammation and atherosclerosis related adipokine, is reduced by glucocorticoids, glucocortidoid therapy may not accelerate atherosclerosis in patients with systemic autoimmune diseases.

## Figures and Tables

**Table 1:** Profile of the 38 patients.

Sex, Male: Female (% female)	11:27 (71.1%)
Age (years)	49.3 ± 15.2
Height (m)	1.61 ± 9.46
Weight (kg)	56.3 ± 11.5
BMI (kg/m^2^)	21.7 ± 3.5
Systemic Autoimmune Diseases:	
SLE (%)	16 (42.1%)
PM/DM (%)	14 (36.8%)
Vasculitis Syndrome (%)	6 (15.8%)
AOSD (%)	2 (5.3%)
Disease Duration (weeks)	4.4 (3.4–12.9)

Data are shown as the number (%), mean ± standard deviation, or median (interquartile range); BMI, body mass index; SLE, systemic lupus erythematosus; PM/DM, polymyositis/dermatomyositis; AOSD, adult-onset Still’s disease.

**Table 2:** Clinical and laboratory data.

	Baseline (*n* = 38)	Follow-up (*n* = 38)	*p* Value
Follow-up period (years)	-	3.3 ± 0.9	-
Comorbidities			
Systolic blood pressure (mmHg)	122 ± 15	124 ± 20	0.399
Diastolic blood pressure (mmHg)	73 ± 10	78 ± 11	0.043
Hypertension (%)	10 (26.3%)	18 (47.4%)	0.185
Diabetes mellitus (%)	4 (10.5%)	9 (23.7%)	< 0.001
Current smoking (%)	11 (28.9%)	4 (10.5%)	< 0.001
Ever smoked (%)	19 (50.0%)	19 (50.0%)	0.607
History of CVD (%)	4 (10.5%)	4 (10.5%)	-
Carotid artery plaque (%)	23 (60.5%)	25 (65.7%)	0.155
Maximum IMT (mm)	0.68 (0.50–0.81)	0.73 (0.59–0.96)	0.043
CAVI	-	7.8 ± 1.5	-
ABI	-	1.2 ± 0.1	-
Laboratory Data			
Total cholesterol (mg/dL)	160 ± 43	198 ± 43	< 0.001
HDL cholesterol (mg/dL)	34 ± 12	69 ± 20	< 0.001
LDL cholesterol (mg/dL)	98 ± 38	105 ± 33	0.304
Triglycerides (mg/dL)	111 (79–186)	102 (70–148)	0.357
CRP (mg/dL)	0.9 (0.2–3.5)	0.1 (0.0–0.33)	< 0.001
Medications			
Daily prednisolone dose (mg)	48.2 ± 9.0 *	7.9 ± 9.9	-
Cumulative prednisolone dose (mg)	0	18651 ± 8734	-
Immunosuppressive agents (%)	0	20 (52.6%)	-
Antihypertensive agents (%)	7 (18.4%)	14 (36.8%)	0.017
Antidiabetic agents (%)	3 (7.9%)	9 (23.7%)	< 0.001
Statins (%)	4 (10.5%)	10 (26.3%)	< 0.001

Data are shown as the number (%), mean ± standard deviation, or median (interquartile range). Significant differences compared with baseline (*p* < 0.05) are underlined; * Initial daily prednisolone dose from just after the baseline examination; IMT, intima-media thickness; CVD, cardiovascular disease; CAVI, cardio-ankle vascular index; ABI, ankle-brachial pressure index; HDL, high-density lipoprotein; LDL, low-density lipoprotein; CRP, C-reactive protein.

**Table 3:** Univariate and multivariate association of clinical data with change of carotid artery IMT.

	ΔCarotid Artery IMT/Year				
	Univariate Model		R^2^	Multivariate Model	
	β	*p* value		β	*p* value
Age	0.001348	0.195	0.046	−0.000477	0.608
Female sex	−0.059111	0.032	0.121	0.034276	0.413
BMI	−0.000186	0.662	0.005	−0.000984	0.790
Cumulative prednisolone dose	0.000003	0.040	0.111	−0.000004	0.011
ΔCRP/year	0.006042	0.233	0.039	−0.014445	0.195
ΔHDL cholesterol/year	0.000069	0.647	0.006	0.000576	0.769
ΔTriglycerides/year	−0.000122	0.123	0.065	−0.000145	0.321
Hypertension	0.028606	0.378	0.022	0.038420	0.153
Diabetes mellitus	0.028536	0.027	0.325	0.074380	0.055
History of CVD	0.034926	0.037	0.022	0.051526	0.186
Ever smoked	0.040737	0.084	0.079	0.009914	0.743
ΔLP/year	−0.027277	0.063	0.093	−0.034033	0.132
ΔRS/year	0.004251	0.157	0.054	0.006317	0.046
ΔHMW-AD/year	0.017032	0.155	0.055	0.008543	0.609
R^2^				0.234	

Significant correlations (*p* < 0.05) are underlined. β, regression coefficient; R^2^, coefficient of determination; IMT, intima-media thickness; BMI, body mass index; CRP, C-reactive protein; HDL, high-density lipoprotein; CVD, cardiovascular disease; LP, leptin; RS, resistin; HMW-AD, high molecular weight-adiponectin.

**Table 4:** Univariate and multivariate association of clinical data with CAVI.

	CAVI (Follow up)				
	Univariate Model		R^2^	Multivariate Model	
	β	*p* Value		β	*p* Value
Age	0.064309	< 0.001	0.554	0.059720	< 0.001
Female sex	−0.582343	0.132	0.064	0.131749	0.733
BMI	−0.015023	0.966	0.000	−0.098105	0.054
Cumulative prednisolone dose	−0.000006	0.718	0.004	-	-
ΔCRP/year	−0.172600	0.551	0.010	-	-
ΔHDL cholesterol/year	0.003258	0.598	0.008	-	-
ΔTriglycerides/year	−0.002384	0.237	0.040	-	-
Hypertension	0.319883	0.446	0.017	-	-
Diabetes mellitus	1.156746	0.038	0.117	1.154566	0.019
History of CVD	0.363258	0.792	0.002	-	-
Ever smoked	0.417251	0.147	0.059	-	-
ΔLP/year	−0.224163	0.321	0.028	-	-
ΔRS/year	0.053863	0.969	0.000	0.088717	0.048
ΔHMW-AD/year	0.339796	0.073	0.089	0.366996	0.122
R^2^				0.539	

Significant correlations (*p* < 0.05) are underlined. β, regression coefficient; R^2^, coefficient of determination; CAVI, cardio-ankle vascular index; BMI, body mass index; CRP, C-reactive protein; HDL, high-density lipoprotein; CVD, cardiovascular disease; LP, leptin; RS, resistin; HMW-AD, high molecular weight-adiponectin.
